# Remarks upon the Therapeutical Action of “Aconite”

**Published:** 1859-07

**Authors:** Jno. R. Cushing

**Affiliations:** South Butler, Alabama


*Remarks upon the therapeutical action of " Aconite."* By JNO. R. CUSHING, M. D., of South Butler, Alabama.

Until I removed to Alabama, some three years since, I was altogether unacquainted with the remedial powers of the " Aconitum Napellus" With the profession in Georgia, " Veratrum Viride" was the great antiphlogistic, and I came with all the prejudices possible, in its favor. I had not only used it with success in many grave cases, but I fear, accredited to it, merits it did not possess. The practitioners in Montgomery, have long since laid it aside, and have been using for several years past, the " Sat. Tinct. of Aconitum." It being a new remedy to me, I studied its powers and action closely, and may say, was rather skeptical for a time—scarcely crediting my own observations. Accident, by some means, threw the pamphlet of the late Dr. Ames, into my hands, on the treatment of Pneumonia with the Aconite—the novelty of the treatment, the high professional character of the doctor, and above all, the statistical result of cases treated, induced me to adopt this mode, and now after two years experience, I can say with satisfaction, I have met with almost uniform success, and I endorse his process as the best I have ever used in the treatment of this disease.

Now, Messrs. Editors, I am no believer in specifics, or pet prescriptions, but to confess the truth I have to acknowledge that the Aconite comes nearer to my notion of being a *specific* than any other agent I have ever used, and with the exception of Mercury, it is my "dernier resort" in every disease in which I come in contact with, when other remedies fail, and many times it supercedes Mercury in its controling influence over diseased action, where the mercurial is contraindicated, or the system will not bear it.

The great embarassment, in the use of "Veratrum" with me, has been, that in many cases, idiocyncrasy forbids its use, and especially in female cases. I have met with mortifying failures, even one drop of the Veratrum producing such irritation as to forbid its use. We all know that "Veratrum" owes its curative effects to arterial sedation, acting upon the system as a nausient, reducing the irritability of the pulse after the same manner of Tart. Emt. and not unfrequently extending its effects upon the bowels, producing irritation, bloody stools etc. Now in regard to the "Aconite" I believe it has a direct curative action through its influence upon the nervous system, independent of its nauseating properties, in small doses, acts as a nervous sedative, also with a direct tendency to relax the tension of the pulse, and in doses large enough to reduce its frequency, its action is pretty much the same as the "Veratrum Viride" with the exception that it is not so often accompanied with intestinal disturbance as that drug, nor do you seldom meet with cases where idiosyncracy forbids its use. I have given it often to children, and seldom meet with a case of the prostration and irritation which so often follows the administration of the "Hellebore," now this is a great advantage of the Aconite, if otherwise it only acted in the same manner as the "Veratrum" and another important item is, that in the use of the Aconite, when arterial excitement has been reduced, it is much more permanent than the reduction produced by the "Veratrum," the doses being given in an inverse ratio, and less frequently, owing, no doubt, to its action upon the brain and spinal marrow, combating directly the diseased state of the nervous system, as the prime cause of disease, or producing a modifying effect ulteriorly upon the heart and arteries, after excitement has once been reduced, and even in subsequent reaction, small doses and repeated less frequently, being all that is necessary in keeping the diseased action, under control, producing not only quiescence in the arterial system, but also in the nervous system.

Dr. Ames* very justly remarks, "that Aconite takes

*See N. O. Medical Journal, January, 1854.

precedence of other remedies that combine properties of a sedative to the heart's action, and of a stimulant to the contractile force of the capillaries; these properties make them, as they have proved to be in practice, especially applicable in all cases of acute disease—applicable in all cases whether acute or chronic, in which the vital powers and force of the heart's action are equal to or above the standard of health. In this class, may be placed, in the order of what I conceive to be, their relative value in acute inflammatory affections generally. * * * * * * Besides its greater efficiency, its application does not require to be limited by any peculiarities in its operation, nor by the character of the organ affected. It is proper to add, in connection with the last remark, that my experience in its use, is limited to inflammation of the brain and its meninges, of the throat, of the lungs and pleura, peritoneum, intestinal mucous membrane, whether attended with dysentery or diarrhœa, rheumatism, chronic or acute, erysipelas, acute corneitis and conjunctivitis.

Dr. Ames' formula in the treatment, is the following, on visiting a patient of adult age, for the first time, laboring under pneumonia, in the first or second stage, pleuro pneumonia, or pneumo bronchitis:

℞.—Tinct. aconitum, nap. (sat.), gtt. xii.
Quinine, sulph. vel. ferro cyan, grs. xxxvi.
Morph. sulph. gr. i.
M. ft. pil., xii.

℞.—Sol. phosphorus, gtt. xvi.
Water, ℥iv. *M.*

Of the first, two pills are directed to be given every third or fourth hour, usually every fourth, each dose being preceded one or two hours, by a teaspoonful of the phosphorus mixture; if an anodyne be required, in addition to that contained in the pills, a quarter of a grain of morphine may be given at bed time; if there be much pain, not yielding permanently to anodynes, a large blister should be applied over the seat of the disease.

A few words, on making and testing the tincture, may not be amiss in this place, and the formula suggested by Dr. Ames, I consider as good as any; you will discover his process is pretty much the same as that given by Dr. Fleming of London. ℞. of the fresh bruised root 1℔, alcohol enough to make one pint of the Tincture. But owing to the different inequalities of the root which we find in our Drug Stores, it should always be tested before used. Dr. Ames "says that the best tincture diluted in proportion to an ounce of water to sixteen drops, taken into the mouth in small quantities, produces a burning in the tongue and lips, with a feeling of tingling and numbness, and a loss of taste, the sensation lasting from two to eight hours."

With myself, there is hardly any disease, in which I do not use this preparation; if there be an exception, it is Typhoid fever. I have never derived much benefit from it in this disease, nor can I explain the reason why; a disease which has baffled our soundest pathologists, and in the treatment of which, the most learned and sagacious of the profession differ, there is no doubt, that acting as a contra stimulant, it depresses too much in this disease where there exists from the beginning a proneness to prostration, and giving way of the vital powers.

But in pneumonia, bronchitis, and all other inflammatory diseases, it supercedes as I have already remarked everything else I ever used, in fact in any disease connected with symptomatic fever, I always find it beneficial, sometimes directly in the beginning of acute attacks, and indirectly in combination with other remedies, in affections of a more protracted nature.

In the treatment of Pneumonia, I generally use it in connection with Phosphorus as suggested by Dr. Ames in the above recipes, there is no doubt but that Phosphorus is an important adjuvant, acting as an expectorant and contra-stimulant of the greatest power. Where blisters have been used to subdue pain, nothing gives more ease than the constant application of large emolient poultices to the blistered surfaces for four or five days. As a matter of course the great alkaloid Quinine should be given all the time in connection with the other remidies. Purgatives, (and especially mercurials) I consider of doubtful utility, unless there be what we call a bilious complication, if such l e the case, as a matter of course a mercurial is necessary, but purgation should be avoided in every instance unless connected with this bilious tendency and then a Blue Pil! and Dovers Powder occasionally, is all that is indicated. Where the liver becomes implicated with inflammation of the base of the right lung, with pain and tenderness on pressure, with vomiting of bilious matter, a few Cups, and a blister will generally prove effectual. The acridness of the alvine discharges, and colicy pains, may be quieted by a few doses of soda and Ess. Pp. menth:

In Pneumonia in this locality, the thing to avoid is that typhoid tendency, the disease is so apt to assume in its second stage, so frequently brought about by the indiscriminate use of purgatives, the course to pursue, is proscribe them altogether, and husband the flagging powers with the remedies already alluded to. If there be foul tongue, delirium, subsultus tendinum, give aconite; if irritability of the stomach, correct portal congestion, and give aconite, use no hurry; if the system tolerates it, use the aconite in the above symptoms as an excitant, not as a depressant; give your quinine and keep your blisters open by poultices, administer your morphine to allay restlessness and quiet nervous agitation, this is the usual course I pursue; there may be many modifications which common sagacity will point out, where deviations may be made. The important landmarks to be kept in view, are to avoid purgatives, use them as seldom as possible; husband the strength and allay irritability, the main idea is, use as little depletion as possible by either the lancet or cathartics.

I have never had much confidence in expectorants in the treatment of the active stages of pulmonary diseases, but when they are used with Phosphorus, there is no doubt but that they are of great efficacy. In regard to phosphorus there is now but little discrepancy of opinion relative to the peculiar influence it exercises over bronchial and other pulmonary diseases, how or in what manner beyond that of a stimulant is hypothetical with me, I only know that it is a master remedy, and when used with the Aconite, an excellent adjuvant in the treatment of Pneumonia.

A few more words on the action of Aconite and I am done. My dose is generally from two to three drops every three or four hours, but the pulse is the great guide of action. When used as an arterial sedative, it is a powerful remedy and sometimes produces alarming effects, and therefore should never be put in the hands of careless nurses, you cannot be too guarded in your directions, for a drop or two, beyond the ordinary dose (tho' not fatal) sometime developes symptoms which is not pleasant to behold. It frequently is desirable to give enough or to repeat the dose often enough to induce some nausea or slight vomiting, particularly in the first stage of Pneumonia, and it is alway safest not to go farther as a general maxim.

There is a point beyond which we cannot go with safety, the symptoms that warn us to stop, are a *peculiar* dryness and coldness in the throat, a difficulty in swallowing etc., and generally complained of by the patient when felt, this you may call "aconitism" and are symptoms that must bid us pause, a few drops beyond this you develope the poisonous action of the drug, which seen once, is sufficient to teach you the caution afterwards, so necessary, in its administration; you will generally find when this coldness and dryness of the throat is complained of, an amelioration in the activity of the disease.

As an expectorant in bronchitis, colds, etc., you can use the following, with great advantage:

℞.—Tinct. aconite, gtt., xxvi.
Tinct. phosphorus, gtt., xvi.
Syr. Tolu., ℥ij. *M.*

Teaspoonful every three hours.

If I had not already run the essay to a tedious prolixity, I might say something in regard to phosphorus, but being in this place irrelevant, I leave it to better hands, and abler pens, to discuss its great merits; however, I may say, I use the anhydrous alcoholic solution.

